# 

**Published:** 1930

**Authors:** 


### The Medical Library of the University of Bristol,

WITH WHICH IS INCORPORATED

**The Library of the Bristol Medico-Chirurgical Society.**

---

*The following donations have been received since the publication of the List in March,* 1930.

*June*, 1930.

| | | |
|---|---|---|
| Hamilton Bailey, Esq., F.R.C.S. (1) | .. .. | 2 volumes |
| Michell Clarke Memorial Fund (2) | .. .. | 1 volume |
| Henry Phipps Institute (3) | .. .. .. | 1 ,, |
| John Purcell Research Memorial (4) | .. .. | 1 ,, |
| Rockefeller Foundation (5) | .. .. .. | 2 volumes |

Unbound periodicals have also been received from Professor E. W. Hey Groves.

### THE ONE HUNDRED AND THIRTY-SECOND LIST OF BOOKS.

The figures in round brackets refer to the figures after the names of the donors. The books to which no such figures are attached have either been bought from the Library Fund or received through the *Journal*.

| | | | |
|---|---|---|---|
| **Bacalogli, C.** | *Clinici Medicale* | | 1929 |
| **Basu, B. D.** | *Diabetes Mellitus and its Dietic Treatment.* 15th Ed. | | 1930 |
| **Fowler, Sir J. K.** | *The Sthenics* | | 1930 |
| **Gould, G. M.** | *Pocket Medical Dictionary* 9th Ed. revised | | 1928 |
| **Hamilton Bailey** | *Branchial Cysts* | (1) | 1929 |
| **Hamilton Bailey** | *Physical Signs in Clinical Surgery* 2nd Ed. | (1) | 1930 |
| **Kittell, P. B.** | *Hæmodynamics* | | 1929 |
| **Lusk, G.** | *Elements of the Science of Nutrition* 4th Ed. | (2) | 1928 |
| **McDougall, J. B.** | *Percussion of the Chest* | | 1929 |
| **MacNevin, M. G., and Vaughan, H. S.** | *Mouth Infections.* Vol. I. | (4) | 1930 |
| **Oliver, W.** | *Practical Essay on Fevers* | | 1704 |
| **Riddell, Lord** | *Medico-Legal Problems* | | 1929 |
| **Rood, F. S., and Webber, H. N.** | *Anæsthesia and Anæsthetics* | | 1930 |

TRANSACTIONS, REPORTS, JOURNALS, ETC.

| | | |
|---|---|---|
| Journal of Industrial Hygiene. Vol. 8 onwards .. .. | | 1926 onwards |
| Medical Annual, 1930 .. .. .. .. .. .. .. .. .. .. .. .. .. | | 1929 |
| Henry Phipps Institute, 21st Report of .. .. .. .. .. .. | (3) | 1929 |
| Methods and Problems of Medical Education. 16th Series .. | (5) | 1930 |
| Quarterly Cumulative Index Medicus. Vol. 6 (July–Dec., 1929) .. | | 1929 |
| Reprints from Methods and Problems of Medical Education on Anatomy and Related Subjects .. .. .. .. .. .. .. .. .. | (5) | 1930 |
| Surgical Clinics of North America. April, 1930 .. .. .. .. .. | | 1930 |
| Transactions of the American Laryngological Association Vol. 51 and Index 1–50 .. .. .. .. .. .. .. .. .. .. .. .. .. .. | | 1929 |

### Local Medical Notes.

**Long Fox Memorial Lecture.**—The lecturer this year will be Professor J. A. Nixon. The subject and date will be announced later.

EXAMINATION RESULTS.

**University of Bristol.**—Students of the University have recently passed the following examinations :—

M.D.—J. F. O. Bodman, T. F. R. Hewer.

Ch.M.—*Pass :* R. V. Cooke.

M.B., Ch.B.—*Second Examination* (*Part II.*). *Pass :* A. V. House, A. D. Jones, M. G. Thomas, S. P. N. Williams.

M.B., Ch.B.—*Final Examination* (*Part I.*). *Pass* (*in Forensic Medicine and Toxicology*) *:* K. P. Beaubrun, L. C. Holland. *In Materia Medica, Pharmacology and Therapeutics and Pathology* (*Completing Examination*). *Pass :* A. J. B. Miall. *In Organic Chemistry* (*Completing Examination*) *:* H. W. Pidgeon.

M.B., Ch.B.—*Final Examination* (*Part II.*). *Pass :* With Second Class Honours, J. F. Coates (with distinction in Medicine and Obstetrics), G. F. Langley (with distinction in Medicine and Public Health). *Pass :* H. F. G. Corfield, R. D. Jones, R. A. Lattey, R. P. Lucas, H. M. Strover. *In Group I.* (*Completing Examination*) *:* R. M. Norman.

D.P.H.—*Part II.* (*Completing Examination*). *Pass :* W. W. S. Sharpe.

B.D.S.—*Third Examination. Pass* (*in Dental Materia Medical, Completing Examination*)*:* M. J. Ryan.

B.D.S.—*Final Examination. Pass :* Muriel E. S. Cosh, W. Forsyth.

L.D.S.—*Preliminary Science Examination. Pass :* J. G. Clancy, R. J. Lee.

L.D.S. — *First Professional Examination. Pass* (*in Physiology and Histology and Anatomy only*)*:* I. R. R. Eskell.

L.D.S. — *Second Professional Examination. Pass* (*in Dental Mechanics and Dental Metallurgy only*)*:* Helena C. L. Blinkworth, W. V. Whiteford. *In Materia Medica only :* R. E. W. Smith.

L.D.S.—*Final Professional Examination. Pass :* A. N. Moon, T. A. Satchwell, C. G. Westlake, I. W. Williams.

CONJOINT.—M.R.C.S., L.R.C.P.—*In Anatomy :* R. H. Moodie. *In Pharmacology and Materia Medica :* Annie M. B. Walker, R. G. Wilbond. *In Pathology :* Anne E. Williams-James. *In Medicine :* Margaret E. Williams, Ida B. Saxby, L. H. Wharton,* R. V. C. Richards, J. F. Coates,* L. H. Griffiths. *In Surgery :* H. M. Strover, J. F. Coates,* A. G. Flemming, C. H. Pauli,* K. I. Nicholls, W. L. Sleight. *In Midwifery :* J. F. Coates.*

L.D.S., R.C.S.—*First Professional Examination. Part I.* (*Dental Mechanics*) *:* J. S. Cochrane, A. M. Watson. *Part II.* (*Dental Metallurgy*) *:* R. H. Green, N. G. Harrison, S. A. McCandlish, P. J. F. Shepherd, H. W. South. *Part IIIa.* (*Anatomy and Physiology*) *:* J. S. Cochrane. *Part IIIb.* (*Dental Anatomy and Physiology*) *:* F. S. Bromley, Margaret D. Hooper, W. N. Trays.

L.D.S., R.C.S.—*Final Examination. Part I. :* G. H. Dakers, T. A. A. Eaves, W. Forsyth, J. K. Noot. *Part II. :* Q. A. Davies, F. G. Lilley, J. P. Price, J. D. Sellers, J. H. White.

* Qualified.

### Publications Received.

---

From JOHN BALE, SONS AND DANIELSSON LTD.:

*The Clinical Pathology of Thoracic Puncture Fluids.* By S. ROODHOUSE GLOYNE, M.D., D.P.H. 1930 Price 10s. 6d. net.

From JONATHAN CAPE LTD :

*The Physiological Principles of Hydrology.* By R. G. GORDON, M.D., D.Sc., F.R.C.P. (Ed.) and F. G. THOMSON, M.A., M.D., F.R.C.P. 1930. Price 5s. net.

From B. FRASER & CO. LTD.:

*The Treatment of Pulmonary and Surgical Tuberculosis with Umckaloabo (Steven's Cure).* By A. SECHEHAYE. 1930. Price 5s. net.

From KEGAN PAUL, TRENCH AND TRUBNER LTD.:

*The Mycoses of the Spleen.* By ALEXANDER GEORGE GIBSON, M.D., F.R.C.P. 1930. Price 12s. 6d. net.

*Diathermy, Medical and Surgical in Oto-Laryngology.* By DAN McKENZIE, M.D., F.R.C.S.E. 1930. Price 10s. 6d. net.

*The Œdema of Bright's Disease.* By CH. ACHARD. 1930. Price 9s. net.

*The Cosmetic Treatment of Skin Complaints.* By E. KROYMAYER. 1930. Price 6s. 6d. net.

From THE LANCET LTD.:

*Guy's Hospital Reports.* January, 1930. Annual subscription £2 2s. or 12s. 6d. net per issue.

From H. K. LEWIS & CO. LTD.:

*Diagnosis and Treatment of Heart Disease.* By E. M. BROCKBANK, M.D. (Vict.), F.R.C.P. 6th Edition. 1930. Price 7s. 6d. net.

*Clinical Examination of Nervous System.* By G. H. MONRAD-KROHN, M.D., F.R.C.P. 5th Edition. 1930. Price 7s. 6d. net.

*A Textbook on the Nursing and Diseases of Sick Children.* By VARIOUS AUTHORS. Edited by ALAN MONCRIEFF, M.D., B.S., M.R.C.P. (Lond.). 1930. Price 15s. net.

*Pharmacopœia of the Central London Ophthalmic Hospital.* 1930. Price 2s. net.

*The Action of Muscles.* By SIR COLIN MACKENZIE, M.D., F.R.C.S., F.R.S. (Edin.). 2nd Edition. 1930. Price 12s. 6d. net.

*The Morphine Habit.* By G. LAUGHTON SCOTT, M.R.C.S., B.A. (Oxon.). 1930. Price 5s. net.

From OXFORD UNIVERSITY PRESS:

*Visceroptosis and Allied Abdominal Conditions associated with Chronic Invalidism.* By H. BEDINGFIELD, D.S.O., M.D. (Ed.), Ch.B., M.R.C.P. (Lond.). 1930. Price 10s. 6d. net.

From THE PANANI OFFICE, BAHADURGANI, ALLAHABAD:

*Diabetes Mellitus and its Dietic Treatment.* By B. D. BASU, I.M.S., Ret. Edited by L. M. BASU, M.B. (Cal.). 15th Edition. 1930. 2 R.s. net.

From THE STUDENT CHRISTIAN MOVEMENT:

*The Doctor's Job.* By A. LLOYD-WILLIAMS, M.B., B.S. (Lond.), M.R.C.S. (Eng.), L.R.C.P. (Lond.). With a Foreword by SIR DONALD MACALISTER OF TARBERT, Bt., K.C.B. 1930. Price 2s. net.

From THE WELLCOME HISTORICAL MEDICAL MUSEUM:

*Henry Hill Hickman. Centenary Exhibition.* 1830–1930. At the Wellcome Historical Medical Museum. 1930.

From JOHN WRIGHT & SONS LTD.:

*Synopsis of Surgery.* By E. W. HEY GROVES, M.S., B.Sc. (Lond.), F.R.C.S. (Eng.). 9th Edition. 1930. Price 17s. 6d. net.

*The Medical Annual.* 1930. Price 20s. net.

*Studies on the Diagnosis and Nature of Cancer.* By VARIOUS AUTHORS. 10s. 6d. net.

*British Journal of Surgery.* April. 1930. Price 12s. 6d. net.

